# Deciphering the driving forces in crystal packing by analysis of electrostatic energies and contact enrichment ratios

**DOI:** 10.1107/S2052252523005675

**Published:** 2023-07-15

**Authors:** Christian Jelsch, Yvon Bibila Mayaya Bisseyou

**Affiliations:** a CRM2, UMR CNRS 7036, Université de Lorraine, Nancy 54500, France; b Laboratoire des Sciences de la Matière, de l’Environnement et de l’Energie Solaire, UFR SSMT, Université Félix Houphouët-Boigny, 22 BP 582 Abidjan 22, Cote d’Ivoire; Sun Yat-Sen University, China

**Keywords:** contact enrichment descriptors, enrichment ratios, electrostatic interaction energy, crystal packing

## Abstract

Hirshfeld surface analysis is a widely used tool for identifying the types of intermolecular contacts that contribute most significantly to crystal packing stabilization. One useful metric for analyzing these contacts is the contact enrichment descriptor, which indicates the types of contacts that are over- or under-represented. In this statistical study, enrichment ratios were combined with electrostatic energy (*E*_elec_) data for a variety of compound families. To compute the electrostatic interaction energy between atoms, charge density models from the ELMAM2 database of multipolar atoms were used. As expected, strong hydrogen bonds such as O/N—H⋯N and O/N—H⋯O typically display large enrichment values and have the most negative (*i.e.* favorable) electrostatic energies. Conversely, contacts that are repulsive from an electrostatic perspective are usually the most under-represented. Analyzing the enrichment ratio and electrostatic energy indicators was shown to help identify which favorable contacts are the most competitive with each other. For weaker interactions, such as hydro­phobic contacts, the behavior is less clear cut and can depend on other factors such as the chemical content of the molecule. The anticorrelation between contact enrichment and *E*_elec_ is generally lost for weaker contacts. However, we observed that C⋯C contacts are often enriched in crystal structures containing heterocycles, despite the low electrostatic attraction. For molecules with only weak hydrogen bond donors/acceptors and hydro­phobic groups, the correlation between contact enrichment and *E*_elec_ is still evident for the strongest of these interactions. However, there are some exceptions where the most favorable contacts from an electrostatic perspective are not the most over-represented. This can occur in cases where the shape of the molecule is complex or elongated, favoring dispersion forces and shape complementarity in the packing.

## Abstract 2

The competition between contacts in crystal structures is statistically analyzed in several families of molecules using electrostatic energy and enrichment ratio descriptors.

## 1. Introduction

The Hirshfeld surface analysis of intermolecular contacts has become a widely used tool to characterize the nature of these interactions (Spackman & McKinnon, 2002 ▸). The Hirshfeld surface partitions the space around a molecule into interior and exterior regions, where the electron density of the promolecule, denoted ρ_int_(**r**), is equal to the electron density issued from the surrounding molecules, denoted ρ_ext_(**r**) (Hirshfeld, 1977 ▸). The surface is representative of the region in space where molecules and atoms interact with each other, and the fingerprint plots generated with the *CrystalExplorer* software (Spackman *et al.*, 2021 ▸) are widely used to describe interactions in crystal packing. More recently, the energy frameworks approach has been developed to aid our understanding of crystal packing by combining accurate intermolecular interaction energies with a graphical representation of their magnitude (Turner *et al.*, 2015 ▸).

This study focuses on decomposing the crystal contacts at the Hirshfeld surface between pairs of interacting chemical species. The derived enrichment ratio *E*_*xy*_ (Jelsch *et al.*, 2014 ▸) provides information on the propensity of chemical species (*X*, *Y*) to form intermolecular interactions with each other. The enrichment ratio is obtained by comparing the actual contact surfaces, denoted *C_xy_*, in the crystal structure with the equiprobable *R_xy_* (random) contacts computed as if all types of contacts had the same probability of forming. The equiprobable contacts are obtained by the probability product *R_xy_* = *S_x_* *S_y_*, where *S_x_* and *S_y_* are the proportions of the species *X* and *Y* contribution on the Hirshfeld surface. The enrichments and contact tendencies were analyzed in several families of halogenated (Jelsch *et al.*, 2015 ▸) and oxygenated (Jelsch & Bibila Mayaya Bisseyou, 2017 ▸) compounds based on their chemical composition.

It was notably observed for molecules containing aromatic cycles with heteroatoms (O, N, halogens) that the C⋯C contacts are over-represented, as denoted by *E*_CC_ enrichments significantly larger than unity. This is due to the possibility of electrostatic complementary of the aromatic surfaces in the interaction, as the presence of heteroatoms generates electron-rich and electron-deficient zones simultaneously in aromatics. The term π⋯π stacking is misleading according to Martinez & Iverson (2012 ▸) and instead shall be called ‘aromatic donor–acceptor interaction’. Conversely, pure hydro­carbon aromatic cycles avoid stacking with each other while T-shaped C—H⋯π interactions are favored as manifested by over-represented C⋯H contacts in this family of compounds. This interaction is attractive from an electrostatic point of view owing to the small charges of opposite signs (C^δ−^⋯H^δ+^) on the two atoms.

In order to statistically analyze the impact of electrostatic attraction/repulsion on the occurrence of contact types in crystal structures, the interatomic electrostatic energies (*E*_elec_) were computed in combination with the contact enrichment in several families of organic compounds. Among molecular interactions, strong hydrogen bonds (N/O—H⋯O/N) involving, notably, ketones, alcohols, carb­oxy­lic acids or carboxyl­ate groups are generally recognized as the most energetically important (Perrin & Nielson, 1997 ▸; Gilli *et al.*, 2004 ▸). C—H⋯O, C—H⋯N and C—H⋯Cl interactions have been shown as weaker hydrogen bonds (Taylor & Kennard, 1982 ▸; Desiraju, 1996 ▸) due to the lower charge of the hydrogen atom bound to carbon. Based on *ab initio* calculations, Gu *et al.* (1999 ▸) suggested that C—H⋯O interactions are true hydrogen bonds. Similarly, less electronegative oxygen atoms in ether, ester and nitro groups for instance also result in weaker O—H⋯O and N—H⋯O hydrogen bonds

Two atoms with significant charges of the same sign display positive electrostatic interaction energy (*E*_elec_). They avoid interaction with each other, resulting in impoverished contacts (*E* ratio smaller than unity). Notably the oxygen atoms, which are generally negatively charged, show low *E*_OO_ enrichment ratios, often close to zero, in the different families of compounds investigated (Jelsch & Bibila Mayaya Bisseyou, 2017 ▸). However, the behavior of atom pairs which show weak attractive or repulsive interactions can be less anticipated. This enrichment/energy meta-analysis investigates some cases where there is competition between several favorable interactions that can form in the crystal packing. In systems with competition between hydrogen bonds, an electrostatic analysis of hydrogen-bond strength enables prediction of the most likely interactions (Aakeröy *et al.*, 2015 ▸).

## 2. Materials and methods

Several families of hydro­carbon molecules containing one or two chemical functions (Table 1 ▸) were searched in the Cambridge Structural Database (CSD, version 5.39; Allen, 2002 ▸) with the software *CONQUEST* (version 1.22, Bruno *et al.*, 2002 ▸). A combination of queries was applied in *CONQUEST* to minimize the number of molecules to be inspected. For example, the criteria to preselect alcohol hydro­carbons were the following: the molecule contains only C, H and O species; contains a C(*sp* ^3^)–O—H group; contains no C=O, C—O—C or COOH groups. Only molecules with 3D structures determined, with no errors, no disorder, no ions (options in *CONQUEST*) and no solvent, were selected. The search was restricted to single-crystal structures with one molecule per asymmetric unit and organometallics were excluded. All CIF structures were checked graphically for correctness and completeness of the hydrogen atoms. Notably, structures with the wrong positioning of the hydroxyl hydrogen atom were detected by high unusual O⋯O or Ho⋯Ho contact enrichment (Jelsch & Bibila Mayaya Bisseyou, 2017 ▸) and were omitted.

The charge density of the molecules was modeled using the multipolar atom of Hansen & Coppens (1978 ▸). The charge density parameters were transferred from the ELMAM2 database of multipolar atoms (Domagała *et al.*, 2012 ▸). Electroneutrality constrain was applied to the molecules after transfer. The *X*—H bond lengths were elongated according to standard neutron diffraction distances (Allen & Bruno, 2010 ▸). The database transfer, the identification of intermolecular contacts and the calculation of *E*_elec_ were performed with the software *MoPro* (Jelsch *et al.*, 2005 ▸).

To take into account only the shortest contacts which contribute to the Hirshfeld surface contacts atom/atom decomposition, a cutoff of the sum of van der Waals radii plus 0.3 Å was applied to the interatomic distances. The average *E*_elec_ value for a given pair of chemical species (*X*, *Y*) was obtained by dividing the summation over all atom pairs by the number of contacts.

The Hirshfeld surface and contact enrichment ratios were obtained with *MoProViewer* software (Guillot *et al.*, 2014 ▸). As *X*⋯*Y* and *Y*⋯*X* contacts yield similar contact surfaces and *E*_elec_ values in the context of this study, the reciprocal contacts were merged together.

## 3. Results and discussion

### 3.1. Aromatic hydro­carbons

**Figure 1:**
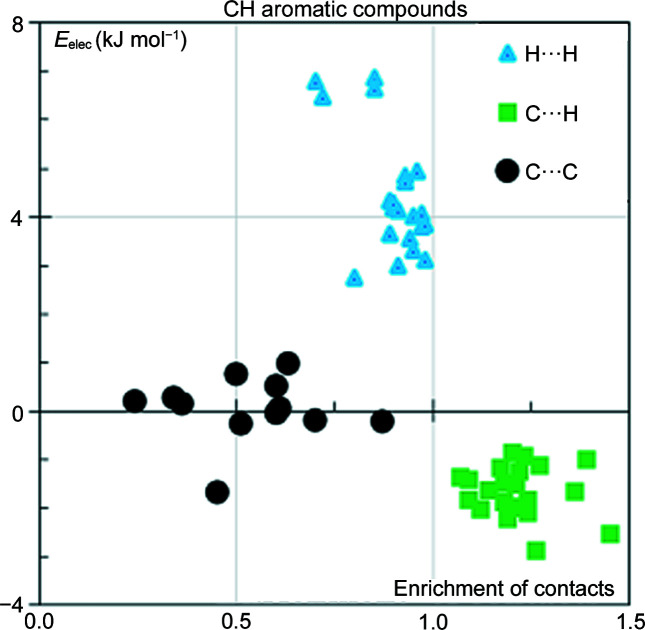
Scatterplot of the electrostatic energy *E*_elec_ of the contact types versus the corresponding enrichment ratios in 22 aromatic hydro­carbons. The *E*_elec_ values are averaged over all contacts of the same type for a given molecule.

Molecules containing a C6 aromatic ring and only hydrogen and carbon atom types were selected. The presence of an aromatic ring here ensures that carbon represents a significant part of the Hirshfeld molecular surface. This type of molecule has only three contact types: C⋯C, H⋯H and C⋯H. A scatterplot of the *E*_elec_ values as a function of the contact enrichment of the molecules is shown in Fig. 1 ▸. The figure clearly shows the clustering of (*E_xy_*, *E*_elec_) points in three zones which confirms the specific role played by each contact type in the crystalline cohesion of aromatic hydro­carbons. The C⋯H contacts are attractive from an electrostatic point of view and are also the only enriched ones. These contacts drive and control crystal packing of molecules in this family which are essentially classified as stack, layer, glide or herringbone (Desiraju & Gavezzotti, 1989 ▸). The H⋯H contacts have enrichment ratios slightly less than unity and have positive electrostatic energy values between +2.5 and +7 kJ mol^−1^. The hydrogen atoms carry positive partial charges which lead to weakly repulsive C^δ−^–H^δ+^⋯H^δ+^–C^δ−^ interactions.

The under-represented C⋯C contacts display electrostatic energy values close to zero, between −1.7 and 1.0 kJ mol^−1^. Though the C⋯C contacts in aromatic hydro­carbons are not repulsive per se, they are not favoured presumably due to competition with the more attractive C—H⋯π weak hydrogen bonds.

### 3.2. Alcohols

**Figure 2:**
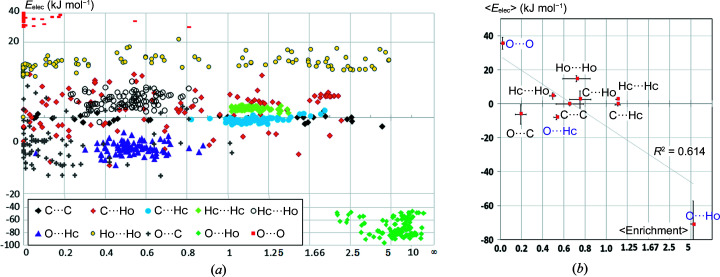
(*a*) Scatterplot of the enrichment and average electrostatic energy for the different contact types in 104 alcohols. For a better visualization of the graph, the abscissa and ordinal axes are not linear. (*b*) Scatterplot of the enrichment and electrostatic energy for the different contact types averaged over all alcohol crystal structures. The error bars represent the sample standard deviations (SSDs). For clarity, enrichment SSDs were divided by 5.

Fig. 2 ▸ shows the electrostatic energy (*E*_elec_) as a function of contact enrichment ratio in a series of 104 alcohol hydro­carbon molecules retrieved from the CSD. Polar Ho hydroxyl hydrogen atoms were distinguished from the more hydro­phobic Hc hydrogen atoms bound to carbon.

The strong O⋯Ho hydrogen bonds form a separate cluster of points in the scatterplot [Fig. 2 ▸(*a*)]. They have, as expected, the largest negative averaged *E*_elec_ energy values in a large range between −46 and −96 kJ mol^−1^ and are correlatively the most-enriched contacts with *E*_O⋯Ho_ values between 2.1 and 21. Conversely the O⋯O and Ho⋯Ho self-contacts are the most repulsive from an electrostatic point of view. The O⋯O contacts in alcohols are strongly avoided with *E* ratios generally close to zero. Ho⋯Ho contacts are not uncommon despite their repulsive nature (*E*_elec_ > 0), as they may occur as a secondary effect of the extensive Ho—O⋯Ho—O hydrogen bonding.

Globally, the two averaged variables 〈*E*〉 and 〈*E*_elec_〉 are anticorrelated with the correlation coefficient *c* = −0.78 [Fig. 2 ▸(*b*)]. When the most attractive O⋯Ho and the most repulsive O⋯O contacts are omitted, the anticorrelation is only *c* = −0.32 for the remaining interactions. This behavior can be understood in the following way: the driving force in the crystal packing stabilization of alcohols resides in the maximization of O⋯Ho hydrogen bonds and in the avoidance of the unfavorable O⋯O contacts. After realization of these two priorities, several weaker contacts do not seem to follow any significant trend with a wide range of enrichment values between 0 and 5. However, the hydro­phobic Hc⋯Hc and C⋯Hc contacts are generally mildly enriched. The weak O⋯Hc hydrogen bonds are weakly attractive and are always present, they are however under-represented at 0.3 < *E*_OHc_ < 0.9 in a limited range of enrichment ratios.

### 3.3. Esters

**Figure 3:**
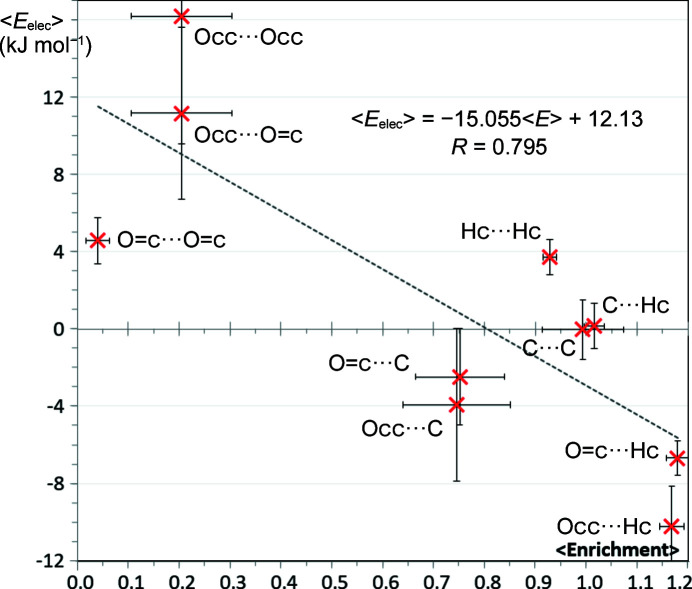
Scatterplot of the average electrostatic energy versus the average contact enrichment ratios for esters. The standard deviations are shown as bars.

A total of 98 molecules composed of ester groups plus aliphatic hydro­carbon fragments were retrieved from the CSD database and analyzed. Two types of oxygen atoms were considered, the O=c atom with one double bond and the Occ atom forming two single bonds with vicinal carbon atoms. Fig. 3 ▸ shows the ten different types of contacts present in this family of compounds which are formed of five types of atoms. Contacts that involve only oxygen atoms (*i.e.* O=c⋯O=c, Occ⋯Occ and O=c⋯Occ) present the most unfavorable electrostatic interactions with electrostatic energy values between +4.6 and +16.2 kJ mol^−1^ and are also the most impoverished (*E* < 0.4). The discrepancy between the three O⋯O contacts in Fig. 3 ▸ has to be relativized to the large standard deviations in both the *E* and the *E*_elec_ values of the latter two contact types.

The most statistically favored contacts of this series are naturally the weak hydrogen bonds of Hc⋯O=c and Hc⋯Occ with similar enrichment ratios of 1.24 and 1.22, respectively. From an electrostatic point of view, the Hc⋯O=c and Hc⋯Occ contacts are the most attractive in this class of compounds. However, Occ⋯Hc contacts appear to have a larger negative electrostatic energy than Hc⋯O=c interactions (the 〈*E*_elec_〉 values are −10.2 and −6.7 kJ mol^−1^, respectively). These values suggest that the Occ oxygen atom is a stronger hydrogen-bond acceptor than the O=c atom within the ester group. Indeed, the ELMAM2 experimental database (Domagała *et al.*, 2012 ▸) used in the present work for the electrostatic energy calculations has a larger number of valence electrons for the Occ oxygen atom (*P*_val_ = 6.198 e) than for the O=c atom (*P*_val_ = 6.138 e). The same trend is also found in the UBDB2018 theoretical database (Kumar *et al.*, 2019 ▸), where *P*_val_(Occ, O202) is 6.193 (25) and *P*_val_(O=c, O103) is 6.116 (34), as well as in the MATTS database (Jha *et al.*, 2022 ▸) where *P*_val_ (Occ) is 6.192 (22) and *P*_val_ (O=c) is 6.112 (34).

The other contacts which are not of O⋯O nor O⋯H type form a third group; they have smaller absolute energy values and have *E* ratios generally not far from unity. Carbon represents on average only 9.9% of the Hirshfeld surface in these aliphatic esters. C⋯C interactions, although having an average electrostatic energy value close to zero (−0.03 kJ mol^−1^) are, on average, slightly over-represented at 〈*E*_CC_〉 = 1.14. Contacts of type Hc⋯Hc with a mean enrichment ratio slightly lower than unity are energetically slightly unfavorable due to partial positive charges (H^δ+^) and undergo competition with O⋯H—C hydrogen bonding.

Note, in this series, some compounds have interaction contacts with atypical enrichment ratios. For example, O⋯O self-contacts, which are electrostatically unfavorable, are, in rare cases, quite over-represented.

**Figure 4:**
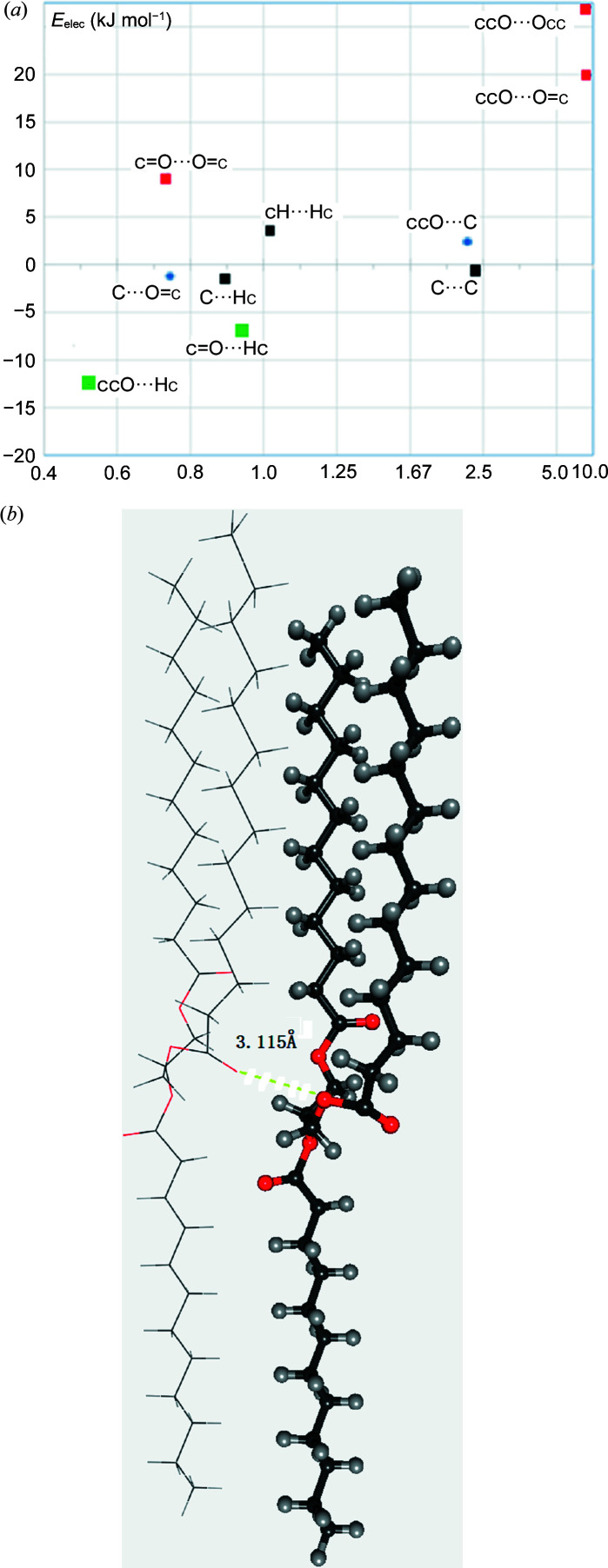
(*a*) Unusual *E*_elec_ versus enrichment scatterplot for the crystal packing of the YIXTUV ester molecule. (*b*) View of YIXTUV molecular interactions.

Fig. 4 ▸(*a*) illustrates the scatterplot (*E*, *E*_elec_) for a compound with the reference code YIXTUV in the CSD (C_41_H_78_O_6_; van Mechelen *et al.*, 2008 ▸). The unfavorable Occ⋯Occ and Occ⋯O=c contacts have very high enrichment ratios of 8.9. Careful inspection of the molecular packing [Fig. 4 ▸(*b*)] shows that Occ⋯Occ and Occ⋯O=c contacts are relatively short with distances of 3.115 and 3.428 Å, respectively, close to 3.04 Å, the sum of the van der Waals radii. Conversely, the favorable Occ⋯Hc and O=c⋯Hc hydrogen bonds are both under-represented at *E* = 0.54 and 0.95, respectively. The YIXTUV molecule is made of three parallel and long aliphatic chains linked by two ester groups. The oxygen content represents only 6.6% of the molecular surface while Hc atoms represent 88%. The unfavorable but enriched O⋯O contacts represent only 1.4% of the contacts which is very small compared with Hc⋯Hc representing 79% of the Hirshfeld surface. Due to the elongated shape and the composition of the molecule, the formation of Hc⋯Hc contacts between the parallel aliphatic chains seems to be the major determinant of the crystal packing formation here.

### 3.4. Chlorinated aromatic hydro­carbons

**Figure 5:**
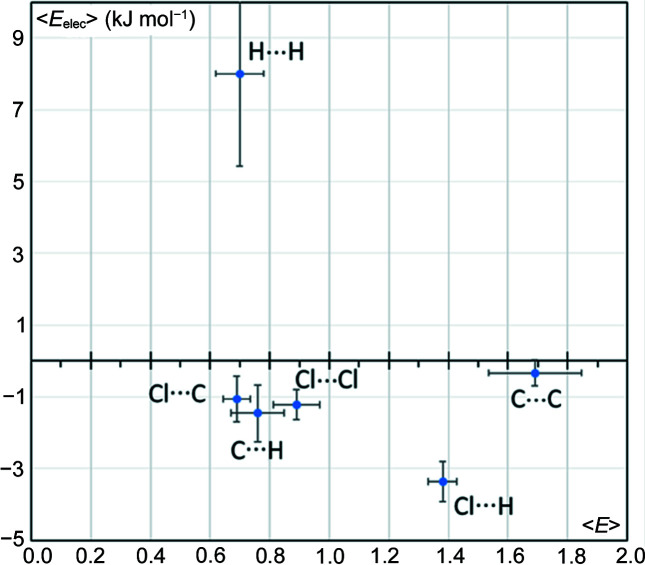
Scatterplot of the average enrichment and electrostatic energy for the different contact types in chlorinated hydro­carbons. The error bars represent the SSDs. For clarity, enrichment SSDs were divided by 5.

This series of purely aromatic molecules is constituted by associations of C6 cycles which are partly chlorinated. Fig. 5 ▸ illustrates the scatterplot of average electrostatic energy (〈*E*_elec_〉) as a function of average contact enrichment ratio (〈*E*〉) in this set of compounds. As can be seen in the graph, H⋯H contacts with 〈*E*_elec_〉 = +8.0 ± 2.6 kJ mol^−1^ are the most energetically unfavorable interactions in this series and they are generally impoverished (〈_⋯_〉 = 0.7 ± 0.4).

On the other hand, the weak Cl⋯H hydrogen bonds are the most attractive contacts with *E*_elec_ in the −1 to −6 kJ mol^−1^ range but are only the second most enriched ones at 〈*E*_ClH_〉 = 1.38 ± 0.24. The attraction of this interaction comes from the partial charges of different signs carried by the two atoms involved in this contact. Indeed, the hydrogen atom on the aromatic ring, which is slightly acidic, is positively charged while the organic chlorine is globally negatively charged. However, the presence of an electropositive σ-hole (Clark *et al.*, 2007 ▸) in the C—Cl bond directions may disfavor the C—Cl^−^⋯H^δ+^ interaction with an angle close to 180°.

To check this hypothesis, a stereochemical analysis was performed on the 234 weak C—Cl⋯H—C hydrogen bonds present in the 29 crystal structures. The frequencies of hydrogen bonds for the different C—Cl⋯H angles are given in Fig. S1 of the supporting information. The hydrogen bonds are clearly under-represented for angles between 150 and 180° where the hydrogen atom is in front of the σ-hole, yet they are over-represented from 90 to 145°, with a maximum around 110°. Interactions with angles below 85° are disfavoured, presumably due to steric hindrance (C⋯H contacts).

A significant correlation (*R* = 0.66) was found between *E*_elec_ and the Cl⋯H distance (Fig. S2) with the shortest interactions being the most attractive. With respect to the C—Cl⋯H angle distribution, the *E*_elec_ values with the largest magnitude are in the 70–130° range (Fig. S3). No correlation (*R* = 0.01) was found between the distance and angle distributions (Fig. S4). The best linear fit of type *E*_elec_ = *f*[(distance, sin(angle-θ)] was found for θ = 14°, yielding a correlation of *R* = 0.73 between the *E*_elec_ values and the fitted ones (Fig. S5). The average value of C—Cl⋯H angles was found to be 118° which is far from linear geometry. Only two C—Cl⋯H interactions out of 234 have angles larger than 160° and their *E*_elec_ energy is among the weakest, less than −2 kJ mol^−1^ in magnitude.

C⋯H contacts, although ranked in second position of energetically favorable interactions (〈*E*_elec_〉 = −1.45 ± 0.79 kJ mol^−1^) are on average impoverished with 〈*E*〉 = 0.76 ± 0.45 obtained on a large range of values from 0.44 to 1.58 (Fig. S6). However, some compounds displaying C—H⋯π interactions have over-represented C⋯H contacts. Compared with Cl⋯H contacts, C⋯H interactions are half as attractive and organic chlorine appears to be a stronger hydrogen-bond acceptor than *sp* ^2^ carbon atoms. Moreover, the carbon atoms bound to chlorine are less prone to form C—H^δ+^⋯π weak hydrogen bonds due to the electron-withdrawing effect of chlorine.

In contrast to the pure hydro­carbon aromatic compounds in which cycle stacking is unfavorable, C⋯C contacts in the chlorinated aromatic rings are generally enriched with 〈*E*〉 = 1.69 ± 0.78, although displaying very small electrostatic energy values in a restricted interval [−0.33, 1.00] kJ mol^−1^ (Fig. S6). The enrichment ratios of C⋯C and H⋯H contacts actually show the strongest anticorrelation with *c* = −0.70 in this class of molecules. The importance of C⋯C interactions in this family is clearly due to the presence of electron-withdrawing chlorine substituents on the carbon atoms. The presence of both electron enriched and depleted carbon atoms provides the cycles with the ability to find favorable electrostatic complementary stacking orientations which are manifested by the settlement of C^δ+^⋯C^δ−^ contacts (Martinez & Iverson, 2012 ▸). Electrostatic effects were found to dominate the trends in interaction energy of the aromatic stacking interactions (Cockroft *et al.*, 2005 ▸).

The Cl⋯C contacts are generally under-represented although they are attractive. The Cl⋯Cl contacts are only slightly under-represented and show a wider range of *E* values between 0.3 and 1.6. Cl^δ−^⋯Cl^δ−^ contacts show weakly negative *E*_elec_ values due to favorable orientations where the σ-hole face of one chlorine atom interacts with the electronegative equatorial torus located around the other (Bui *et al.*, 2009 ▸; Hathwar & Row, 2010 ▸). The electrostatic energy between two neutral spherical atoms is negative. Due to this effect, two atoms with weak charges of same sign at around van der Waals contact distance can still display attractive electrostatic interaction. For instance, in the compound with the reference code FECZOE (Li *et al.*, 2012 ▸), there is a Cl⋯Cl contact at a distance of 3.884 Å and the penetration energy (electrostatic between two neutral spherical atoms) is −0.75 kJ mol^−1^.

The chloro­benzene compound (MCBENZ; André *et al.*, 1971 ▸) has a crystal structure among the most enriched Cl⋯Cl contacts (*E* = 1.6) and displays chains of interacting chlorine atoms along the *b* axis (Fig. S7). For the compound C_6_Cl_5_, a Cl_3_ synthon is observed in the crystal packing (Brezgunova *et al.*, 2012 ▸) and the Cl⋯Cl contacts are also slightly enriched (*E* = 1.09).

To summarize, in view of the (*E*_elec_, *E*) scatterplot for chlorinated aromatic hydro­carbons, the packing seems primarily ruled by the realization of weak C—H⋯Cl hydrogen bonds and of aromatic stacking. All other contact types are, on average, moderately under-represented, notably H⋯H, which is the least favorable contact in terms of electrostatics.

### 3.5. Amide compounds

Hydro­carbons containing amide groups were investigated as they contain both strong hydrogen-bond donors and acceptors. The presence of a strong proton acceptor and donor intuitively gives rise to the formation of hydrogen bonds during self-assemblies. It has been observed that amide compounds interact with each other, forming the N—H⋯O=C hydrogen bond called an ‘inter-peptide hydrogen bond’ which controls and stabilizes the supramolecular assembly (Dixon *et al.*, 1994 ▸). This hydrogen bond type is widely recognized as being largely responsible for the secondary and tertiary structure of proteins in α-helices and β-sheets.

**Figure 6:**
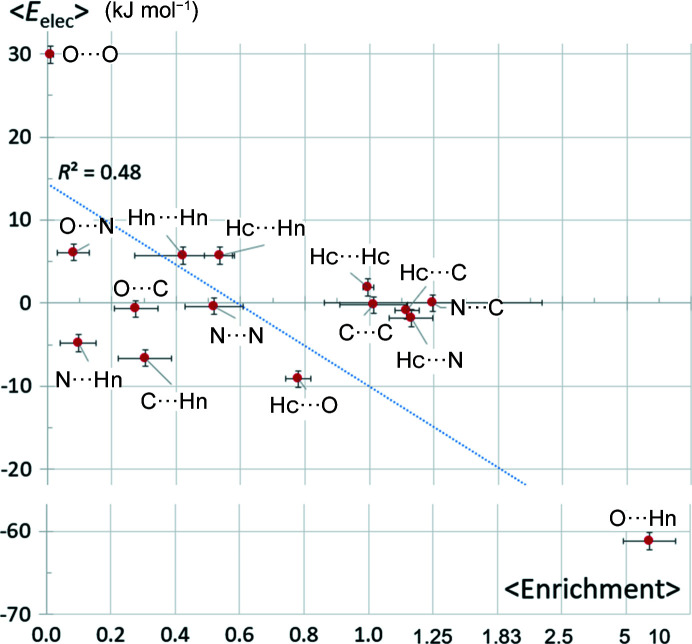
Scatterplot of the average electrostatic energy versus the average contact enrichment ratios for 69 amide molecules. Horizontal error bars correspond to the SSDs divided by 5.

Fig. 6 ▸ displays the (〈*E*〉, 〈*E*_elec_〉) scatterplot of interatomic contact types of a 69 amide compound series retrieved from the CSD. The point associated with the O⋯Hn strong hydrogen bond is clearly distinguished from the others. This ‘inter-peptide hydrogen-bond’ is, by far, electrostatically the most stable with an average *E*_elec_ value of −61 ± 12 kJ mol^−1^ and is also the most enriched (〈*E*〉 = 7.9 ± 5.8). There are, however, outlier molecules with zero enrichment of the O⋯H—N hydrogen bond. The C_30_H_48_N_4_O_4_ molecule with the reference code OJICAL has four amide bonds which form four internal O⋯H—N interactions; these are not counted in the contacts at the Hirshfeld surface. The oxygen atoms form intermolecular C—H⋯O hydrogen bonds (*E* = 1.07) with a large number of methyl groups present in the molecule. Another outlier, the RODJUP molecule (Fig. S8), has only one amide moiety but as many as four phenyl groups. Its packing is devoid of strong hydrogen bonds due to the difficulty for such a bulky molecule to form an N—H⋯O hydrogen bond.

The weak hydrogen bond O⋯Hc is, on average, the second most energetically attractive contact of the series, but it is very slightly depleted at 〈*E*〉 = 0.8 ± 0.2, presumably due to the competition of the strong O⋯Hn hydrogen bonds.

The self-contacts of charged atoms are unprivileged and are electrostatically disadvantaged. The hydro­phobic atoms C and Hc, whose self-contacts have small electrostatic energy values, are normally represented (*E* = 1.0). The C⋯C contacts are not privileged here, in contrast to the case of halogenated aromatics, as the aromatic rings (when present) are not substituted. Among the self-contacts, the O⋯O interaction is the most impoverished and the most repulsive [〈*E*_elec_〉(O⋯O) = +29.9 ± 1.5 kJ mol^−1^] followed by the Hn⋯Hn contact with an average electrostatic energy value of +5.7 ± 0.7 kJ mol^−1^.

The weak contacts, with |〈*E*_elec_〉| < 10 kJ mol^−1^ in magnitude, show a large variety of mean contact enrichment ratios between 0.1 and 1.1, with no clear correlation between the two variables. The hydro­phobic lowly charged Hc^δ+^ atoms, which are generally abundant on the molecular surface, in fact display slightly enriched contacts with the lowly negatively charged N amide atoms (*Q*_val_ = −0.14 e) and with the (non-amide) C^δ−^ atoms.

### 3.6. Pyridine + amide compounds

**Figure 7:**
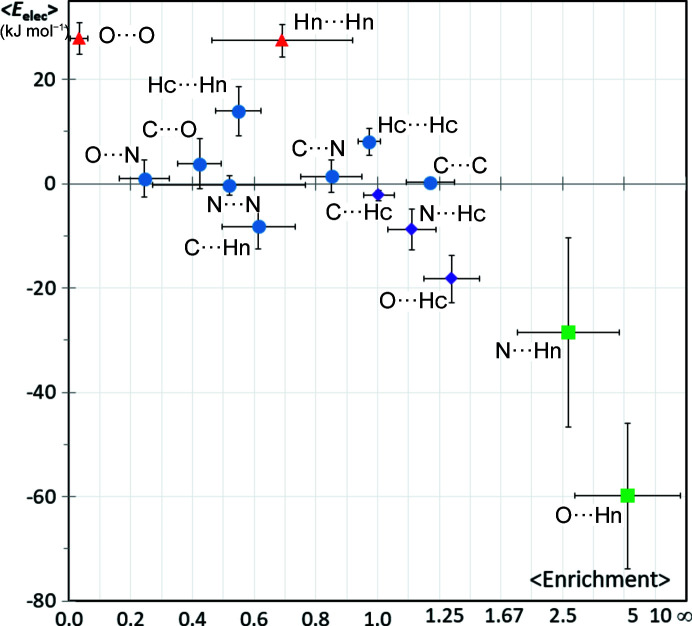
Scatterplot of the average *E*_elec_ versus the average contact enrichment ratios for hydro­carbons containing both amide and pyridine groups. The SSDs are shown as error bars. For the abscissa axis, the SSDs are divided by 5 for clarity. Two Hn⋯Hn contacts were omitted due to non-meaningful values of enrichment resulting from very small *S*_Hn_ (<2.4%) and *R*_HnHn_ values.

In order to analyze the competition between two strong hydrogen-bond acceptors, hydro­carbons containing both an amide carbonyl and a nitro­gen with a lone pair within a C_5_N aromatic cycle were retrieved from the CSD. These molecules have the O=C—NH_2_ amide group with two strong hydrogen-bond donors. The scatterplot of the average electrostatic energy 〈*E*_elec_〉 as a function of average contact enrichment ratio of interatomic contact types of this series is illustrated in Fig. 7 ▸. The distribution of points shows some similarity with the amide series. The O⋯Hn hydrogen bond is the most enriched and attractive contact, while the O⋯O contact type is the most impoverished and repulsive in both series. However, the O⋯Hn contact is less enriched compared with the amide series (〈*E*〉 = 5.2 here versus 8.4) due to competition of the other strong hydrogen bond present, namely the N⋯Hn interaction.

The O⋯Hn hydrogen bonds appear to be twice as attractive than N⋯Hn (〈*E*_elec_〉 = −60 ± 14 versus −28 ± 18 kJ mol^−1^) which is also reflected in the higher enrichments (5.2 versus 2.6).

Analysis of the pyridine/amide compound family via both *E* and *E*_elec_ descriptors confirms that the oxygen atom of the amide carbonyl functional group is a stronger hydrogen-bond acceptor site compared with the *sp* ^2^ nitro­gen atom with the electron lone pair. The donor⋯acceptor (*D*⋯*A*) distances were found in a CSD survey by Kuleshova & Zorkii (1981 ▸) to be on average 0.1 Å shorter for C=O⋯H—N hydrogen bonds compared with >N⋯H—N.

A number of other oxygen acceptors, such as ethers, are however weaker donors than the >N nitro­gen atoms with a lone pair. For instance, Nobeli *et al.* (1997 ▸) surveyed the CSD for hydrogen bonds between alcohol donors and aromatic fragments containing one or more nitro­gen and/or oxygen acceptor. They found that hydrogen bonds to nitro­gen atoms are much more abundant than to C—O—C and C—O—N type oxygens; these results were corroborated by energy calculations.

Böhm *et al.* (1996 ▸) compared the propensity of a water hydrogen-bond donor to interact with nitro­gen Ncc and oxygen Occ atoms. Based on interaction energies obtained from *ab initio* calculations for complexes of these molecules with water, the oxygen atoms can be classified as weaker hydrogen-bond acceptors than the nitro­gen atoms. For the amide + pyridine class of compounds, the three weak and two strong hydrogen bonds show enrichment ratios that remarkably follow the strength of the electrostatic energy in this order: C⋯H—C < N⋯H—C < O⋯H—C < N⋯H—N < O⋯H—N.

### 3.7. Chlorinated ethers

**Figure 8:**
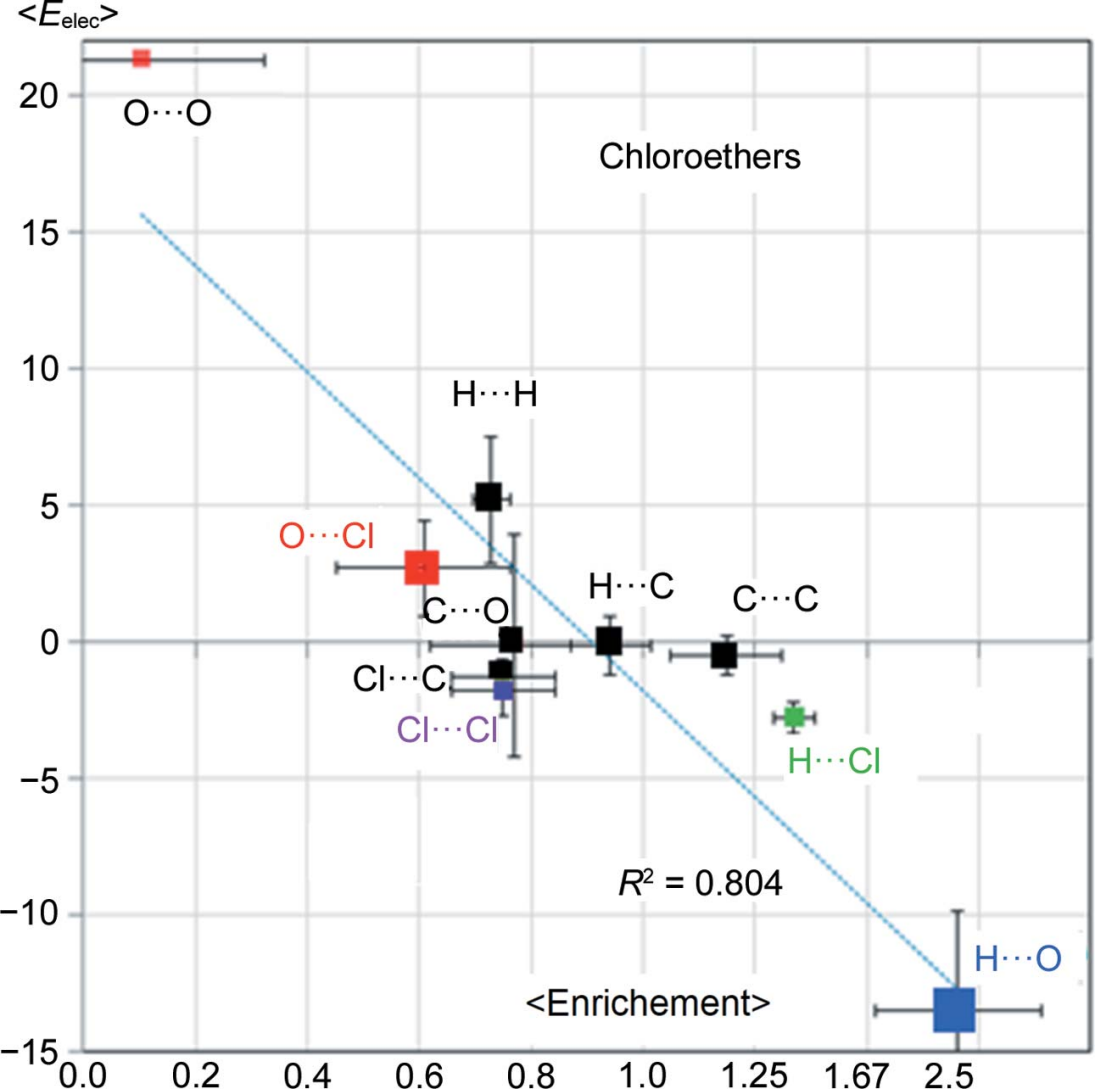
Scatterplot of the average *E*_elec_ versus the average contact enrichment ratios for chlorinated hydro­carbons containing an ether group. The SSDs are shown as error bars. For the abscissa axis, the SSDs are divided by 5 for clarity.

Exploration of compounds in the chlorinated ether family offers an opportunity to analyze the behavior of organic chlorine in the presence of the ether oxygen, both chemical functions being weak hydrogen-bond acceptors. The average electrostatic energy as a function of average contact enrichment ratio on a set of molecules retrieved from the CSD is plotted in Fig. 8 ▸. This class of compounds has the possibility to form two types of weak hydrogen bonds, C—H⋯O and C—H⋯Cl and the very weak C–H⋯C hydrogen bond. In addition, the so-called Cl⋯O halogen bond (Metrangolo & Resnati, 2001 ▸, 2014 ▸; Cavallo *et al.*, 2016 ▸) may also form. The weak hydrogen bonds, H⋯O followed by H⋯Cl, are the most attractive interactions with the largest negative values of electrostatic energy. These two most attractive contacts correspond also to the most enriched ones, in the same order.

The Cl⋯O halogen bonds here appear to be generally slightly repulsive interactions with 〈*E*_elec_〉 = +2.7 ± 1.7 kJ mol^−1^, which is corroborated by their moderate impoverishment with an average 〈*E*〉 = 0.61. The extremal *E*_elec_ values are −1.2 and 4.3 kJ mol^−1^. The only compound in the series with a negative electrostatic energy between the oxygen and chlorine atoms in contact is EBENIL (Joshi *et al.*, 2011 ▸; Fig. S9); the geometry is *d*(O,Cl) = 2.93 Å and the angle C—Cl⋯O = 164.7°, not far from 180°, allows the electropositive σ-hole of the Cl atom to interact with the oxygen lone pairs. It has to be recalled that the ether oxygen atom that is in an *sp* ^2^ carbon environment, as in the case of the EBENIL molecule, has two oxygen lone pairs which appear merged in deformation electron density maps (Ahmed *et al.*, 2013 ▸).

The low attractiveness of the O⋯Cl halogen bonds and even their repulsive character in this series of compounds is related to the slight electronegativity of the two atoms. Dikundwar *et al.* (2014 ▸) observed in the crystal structures of a series of halo­fluoro­benzenes that fluorine favors hydrogen bonds over halogen bonds. When the electropositive σ-hole on the chlorine atom faces the oxygen atom however, the interaction is more favorable. The repulsive effect is reinforced when the chlorine atom is in an electron-donating environment. However, when the environment around the halogen atom is sufficiently electron-withdrawing, the resulting halogen bond can become more favorable and occurs more frequently in crystal packings (Amico *et al.*, 1998 ▸).

The 〈*E*〉 ratios as well as the 〈*E*_elec_〉 values for the H⋯O and H⋯Cl contacts confirm the much stronger hydrogen-bond acceptor character of oxygen compared with organic chlorine. When competing in the formation of attractive interactions, the H⋯O weak hydrogen bonds are the main driving force in crystal packing stabilization for the chloro-ether series.

The most repulsive interaction is the O⋯O self-contact at 〈*E*_elec_〉 = +21.3 kJ mol^−1^ and has the lowest contact enrichment 〈*E*〉 = 0.10 ratio. The graph (〈*E*〉, 〈*E*_elec_〉) shows a significant global anticorrelation *r* = −0.90 for the chloro-ethers. When the two interactions (O⋯O and H⋯O) with the highest energy magnitude are omitted from the (〈*E*〉, 〈*E*_elec_〉) linear fit, the correlation to the determination coefficient drops to *r* = −0.56.

The H⋯H contact is the second most unfavorable with 〈*E*_elec_〉 = +5.2 kJ^−1^ and is mildly impoverished at 〈*E*〉 = 0.73. The Cl^δ−^⋯Cl^δ−^ contacts display small attractive 〈*E*_elec_〉 values due to the presence of the electropositive σ-hole on the negatively charged chlorine atoms (Bui *et al.*, 2009 ▸). The contact appears relatively impoverished at 〈*E*〉 = 0.75, presumably due to competition with the slightly more favorable Cl⋯H hydrogen bond. The C⋯C interaction, with an *E*_elec_ energy value close to zero, appears slightly enriched at 〈*E*〉 = 1.15, as was observed in the previous series of molecules with the exception of the pure hydro­carbons.

### 3.8. Fluorinated nitrile hydro­carbons

**Figure 9:**
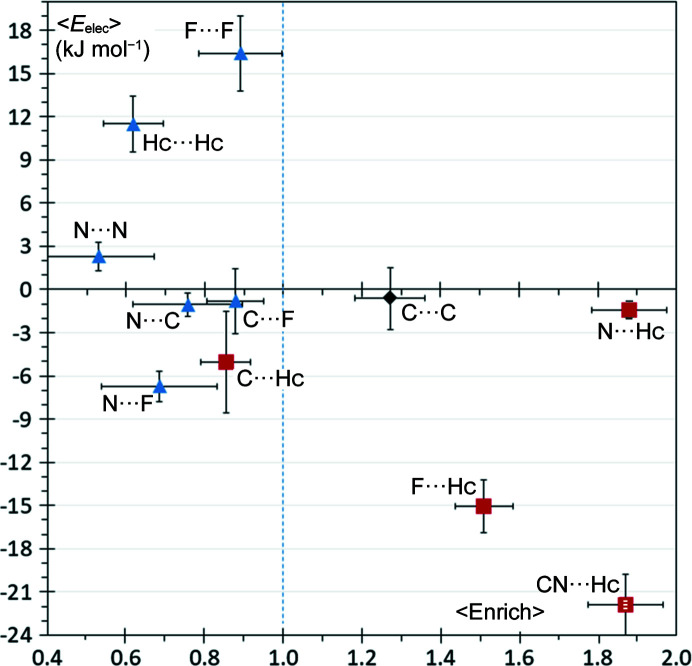
Scatterplot of the average *E*_elec_ versus the average contact enrichment ratios for hydro­carbons containing both fluorine and nitrile groups. The SSDs are shown as error bars. For the ordinate axis, the SSDs are divided by 2 for clarity. For the N⋯Hc contact, two symbols are shown, using the energy between N and Hc atoms and between the whole CN moiety and Hc, the enrichment ratio corresponding to the N⋯Hc contact.

In this series of compounds, organic fluorine is an electron-withdrawing atom and a weak hydrogen-bond acceptor. The N⋯Hc and F⋯Hc contacts, which are both weak hydrogen bonds, are distinguished from the other contacts with enrichment ratios significantly greater than unity (〈*E*〉 = 1.9 ± 0.4 and 〈*E*〉 = 1.5 ± 0.3, respectively; Fig. 9 ▸).

The average electrostatic contribution between N and Hc atoms in such hydrogen bonds is only −1.9 ± 1 kJ mol^−1^. On the other hand, the mean *E*_elec_ value between the neighboring nitrile carbon N and Hc is −20 ± 4 kJ mol^−1^. This is related to the valence population-derived atomic charges (*Q* = *N*_val_ − *P*_val_) which take the values *Q*(N) = +0.06 e and Q(C) = −0.26 e when derived from the valence populations in the ELMAM2 database. The deformation electron density of the CN group is shown in Fig. S10. The nitro­gen atom of the CN group acceptor has an electron lone pair and is a hydrogen-bond acceptor, but its electrons are partially withdrawn towards the electronegative carbon atom, as seen in the valence populations. Despite the electron-withdrawal, the electrostatic potential takes the most negative values in the nitro­gen lone pair region (Fig. S11).

As most of the electrostatic energy in the CN⋯Hc hydrogen bonds comes from the C and Hc atoms, we decided to also compute the energy for the whole CN group for this particular interaction. By grouping the CN atoms, the CN⋯Hc contact appears to be the most energetically attractive contact and it is followed by the F⋯Hc contacts. The CN⋯Hc hydrogen bonds have, on average, *E*_elec_ values 30% larger than the F⋯Hc ones with 〈*E*_elec_〉(CN⋯Hc) = −22 ± 4 kJ mol^−1^ and 〈*E*_elec_〉(F⋯Hc) = −15 ± 4 kJ mol^−1^. The enrichment ratio results confirm that the nitrile group is a stronger hydrogen-bond acceptor than organic fluorine.

The F⋯F self-contacts between charged species are the most energetically unfavorable and are generally under-represented. Indeed, fluorine bears a negative charge and the σ-hole is much weaker compared with other halogen atoms (Metrangolo *et al.*, 2011 ▸). As a result, the F⋯F contacts with the best orientation when the σ-hole of one atom faces the equatorial electronegative torus of the other still have positive *E*_elec_ values around +7.3 kJ mol^−1^ in the sample of molecules. The preference seen in Fig. 9 ▸ of fluorine for hydrogen bonds over halogen bonds (such as F⋯F) is in accordance with investigations by Dikundwar *et al.* (2014 ▸). Hc⋯Hc self-contacts with 〈*E*_elec_〉 = +11 ± 4 J mol^−1^ are second with respect to average electrostatic repulsion and are generally quite unprivileged with 〈*E*〉 = 0.62 ± 0.25.

The C⋯Hc contacts, although energetically attractive, on average, show a large range of individual energy values between −26 and +26 kJ mol^−1^ due to the presence of negatively charged nitrile carbon atoms and positively charged atoms bound to fluorine in this class of compounds. The C⋯Hc interactions, corresponding mostly to very weak C—H⋯π hydrogen bonds, are presumably under-represented due to competition with the N⋯Hc and F⋯Hc hydrogen bonds.

C⋯C contacts with disparate small average electrostatic energy values of 〈*E*_elec_〉 = −0.6 ± 4 are the only enriched interactions at 〈*E*〉 = 1.27 ± 0.37, aside from the N⋯Hc and F⋯Hc hydrogen bonds. These results confirm previous findings that, in compounds with substituted aromatic rings, C⋯C contacts are enriched due to the possibility of favorable electrostatic complementary stacking orientations (Salonen *et al.*, 2011 ▸; Martinez & Iverson, 2012 ▸; Jelsch *et al.*, 2014 ▸).

### 3.9. ROY polymorphs

**Figure 10:**
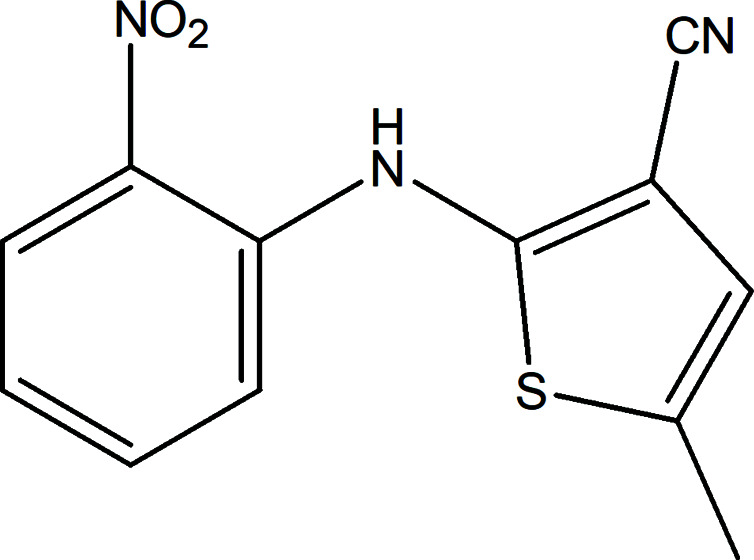
2D structure of ROY molecule. The different conformational polymorphs involve changes in the C—N—C—S dihedral angle.

**Figure 11:**
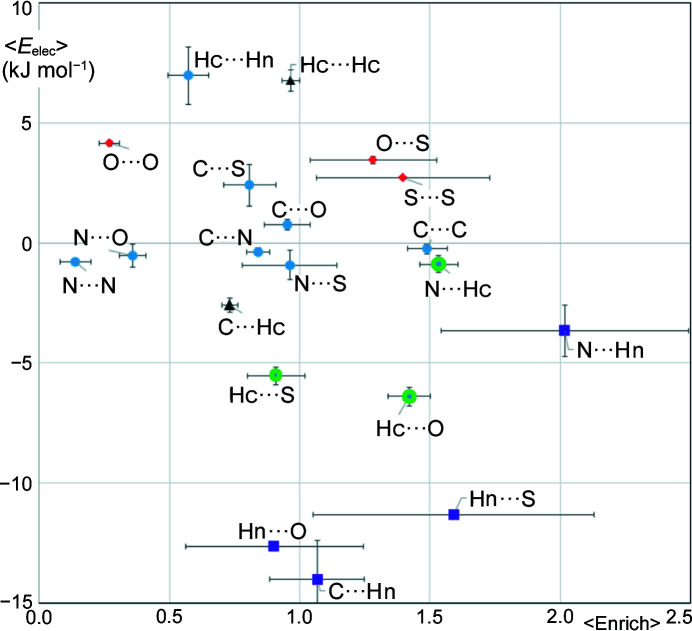
Scatterplot of the average enrichment and average electrostatic energy for ten different polymorphs of the ROY molecule. Standard deviations on enrichment and *E*_elec_ have all been divided by 5.

At present, the ROY molecule, 5-methyl-2-[(2-nitro­phenyl)amino]-3-thio­phene­carbo­nitrile (Fig. 10 ▸), has the largest number of crystalline polymorphs of any molecular system recorded in the CSD (Galek *et al.*, 2009 ▸; Lévesque *et al.*, 2020 ▸). With this singularity, the ROY molecule is an ideal molecular system for testing computational models as stipulated by Yu (2010 ▸). The ROY molecular structure contains three potential hydrogen-bond acceptor groups (–NO_2_, –CN and >S), only one strong N—H donor and eight weak C—H donors. The strength of the N—H donor is attenuated by the fact that it is already involved in an intramolecular N—H⋯O hydrogen bond with the nitro group. It has to be recalled that the carbon atom of the nitrile group is more electronegative (*P*_val_ = 4.12 e) in the ELMAM2 database than the nitro­gen atom (*P*_val_ = 5.00 e) which bears an electron lone pair. Fig. 11 ▸ illustrates a detailed scatterplot of average electrostatic energy as a function of average contact enrichment ratio in ten ROY polymorph crystal structures available in the CSD.

The most attractive contacts are the hydrogen bonds NC⋯Hn, O⋯Hn and S⋯Hn with energy values in the range −14 to −11 kJ mol^−1^. Among these hydrogen bonds, only S⋯Hn is significantly enriched at 〈*E*〉 = 1.59. On the other hand, the CN⋯Hn hydrogen bond is less attractive but is the most over-represented contact at *E* = 2.02. The O⋯H—N, S⋯H—N and N⋯H—N hydrogen bonds are each present in one polymorph, while the C⋯H—N hydrogen bond occurs in five polymorphs. The N—H group is not involved in an intermolecular hydrogen bond and interacts only with carbon and hydrogen atoms in the polymorphs R05 (two molecules per asymmetric unit) and ORP.

The hydrogen-bond acceptors in ROY that do not interact with the strong N—H donor have the possibility to form hydrogen bonds with the weak C—H donors. The interactions of C—H with oxygen, sulfur and nitro­gen are moderately attractive and are well represented, notably N⋯Hc and O⋯Hc are significantly enriched (*E* = 1.54 and 1.41, respectively).

In the ROY series, there is the small numbers statistical effect; as there is a large number of different atom types, including several acceptors, a given contact type is often incidentally absent. The Hc⋯Hn and Hc⋯Hc are the most repulsive contacts occurring in the ROY polymorphs while the even more unfavorable Hn⋯Hn contact is completely absent. The Hc⋯Hc contacts are however normally represented with an average enrichment ratio close to unity. As for O⋯O self-contacts, they are avoided (〈*E*〉 = 0.24 ± 0.20) and are energetically unfavorable (〈*E*_elec_〉 = 4.1 ± 0.6 kJ mol^−1^) as observed in other series of oxygenated compounds analyzed.

The C⋯C interactions follow the typical behavior observed in several families of compounds. Indeed, the C⋯C stacking contacts with an electrostatic energy average value close to zero are significantly enriched contacts at 〈*E*〉 = 1.41 ± 0.30. ROY has a phenyl ring substituted by a nitro group and a thio­phene-ring with nitrile and methyl substituents. The possibility to have an electrostatic complementarity between these heterocycles in stacking interactions results in a significant enrichment of the C⋯C contacts. Only one polymorph (ORP) has under-represented C⋯C contacts with *E* = 0.84 while the highest enrichment reaches 1.88 for the OP polymorph.

## 4. Conclusions

The electrostatic energy is a widely recognized and powerful metric to evaluate the strength and stability of interatomic interactions within molecular complexes and crystal structures. In this paper, we present an analysis of intermolecular contact interactions in a variety of chemical systems in their crystalline state. To accomplish this, we employ a novel approach that combines the calculations of electrostatic interaction energies, derived from the multipolar atom model, with the contact enrichment ratio. We have retrieved compounds from the CSD and conducted our analysis using this methodology.

This study reveals several important observations. The findings suggest that strong hydrogen bonds, such as O/N—H⋯O/N, are highly attractive and have large contact enrichment ratios (greater than 5, up to 15). These hydrogen bonds are the primary contribution to the electrostatic stabilization energy of the crystal packing for compounds that have strong hydrogen-bond acceptors and donors. Compounds that have strong hydrogen-bond acceptors but lack strong donors (such as ketone hydro­carbons and esters) exhibit moderately enriched weak C—H⋯O hydrogen bonds.

Self-contacts between charged atoms yield positive *E*_elec_ values. O⋯O contacts are generally repulsive interactions and are under-represented in many types of molecules. A similar trend is observed for H⋯H contacts, including Ho⋯Ho and even Hc⋯Hc, which is an atom type with a smaller charge.

However, there are exceptions to the above observations. For instance, in crystals where molecular shape plays a more significant role in achieving shape complementarity in crystal packing, there may not be a significant anticorrelation between *E*_elec_ and the enrichment ratio.

In the case of ten ROY polymorphs, the analysis revealed a weak anticorrelation (*r* = −0.28) between the average electrostatic interaction energies and enrichment ratios. The ROY molecule, with four hydrogen acceptors and only one strong donor, results in the formation of a maximum of one strong hydrogen bond among four possibilities and in several weak hydrogen bonds involving C—H groups.

This meta-analysis indicates that weak contacts in crystal structures do not always exhibit a clear trend, even after the most attractive contacts are formed and the most repulsive are avoided. However, for compounds with a small number of chemical types, weak contacts can still follow certain patterns. For instance, weak C—H⋯O hydrogen bonds in alcohols are typically present but moderately under-represented, as they are much less attractive than O—H⋯O interactions.

Interestingly, the C⋯C contact type exhibits a peculiar behavior. Although C⋯C contacts have an electrostatic energy close to zero, they appear quite frequently over-represented in compounds with heterocycles, which allow for aromatic stacking with electrostatic complementarity (Salonen *et al.*, 2011 ▸; Jelsch *et al.*, 2014 ▸). The large contact surface in aromatic stacking may also result in other attractive energy contributions, such as dispersion, playing an important role.

The electrostatic energies (*E*_elec_) were computed here between pairs of interacting atoms on the basis of pseudo-atom superposition modeling the molecular electron densities after multipolar database transfer. It would be interesting to carry out a similar (*E*, *E*_elec_) study using interaction energies obtained directly from quantum mechanics, for example with the Interacting Quantum Atoms (IQA) method (Menéndez Crespo *et al.*, 2021 ▸).

## Supplementary Material

Supporting figures. DOI: 10.1107/S2052252523005675/yc5043sup1.pdf

## Figures and Tables

**Table 1:** Families of hydro­carbon molecules studied

Family	Number	Chemical species
Aromatic hydro­carbons	22	C H
Alcohols	104	C H O
Esters	98	C H O
Chlorinated C6 aromatics	29	C H Cl
Amides	69	C H N O
Amides + pyridines	71	C H N O
Chlorinated ethers	51	C H O Cl
Fluorinated nitrile hydro­carbons	19	C H N F
ROY polymorphs	10	C H N O S
