# Supplementary material for: Deciphering the driving forces in crystal packing by analysis of electrostatic energies and contact enrichment ratios
Source: IUCrJ. 2023 Jul 15;10(Pt 5):557–67. doi: 10.1107/S2052252523005675 (PMC10478513; doi:10.1107/S2052252523005675)
Supplement: Supplementary file 1 [file m-10-00557-sup1.pdf]

IUCrJ

Volume 10 (2023)

Supporting information for article:

Deciphering the driving forces in crystal packings by analysis of electrostatic energies and contact enrichment ratios

Christian Jelsch and Yvon Bibila Mayaya Bisseyou

Supplement IUCr J. 2023

Deciphering the driving forces in crystal packings by analysis of electrostatic energies and contact enrichment ratios

Christian Jelsch & Yvon Bibila Mayaya Bisseyou

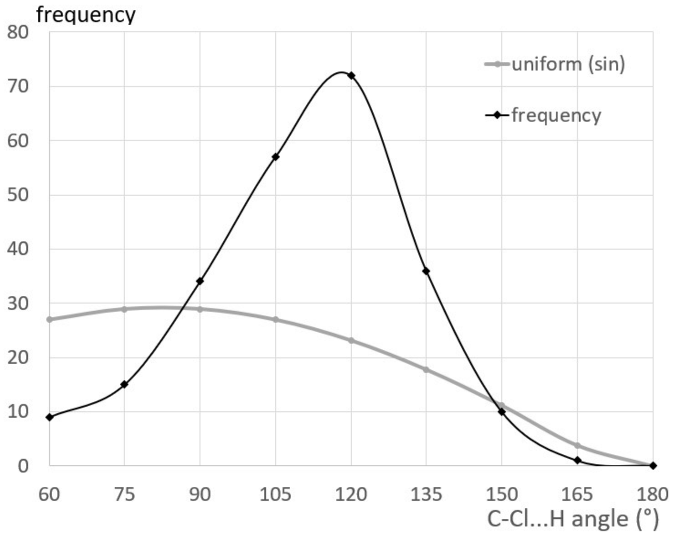

Figure S1. Occurrences of 234 C–Cl…H interaction angles in the chlorinated aromatic hydrocarbons. The grey curve represents the frequencies for a uniform distribution in all directions.

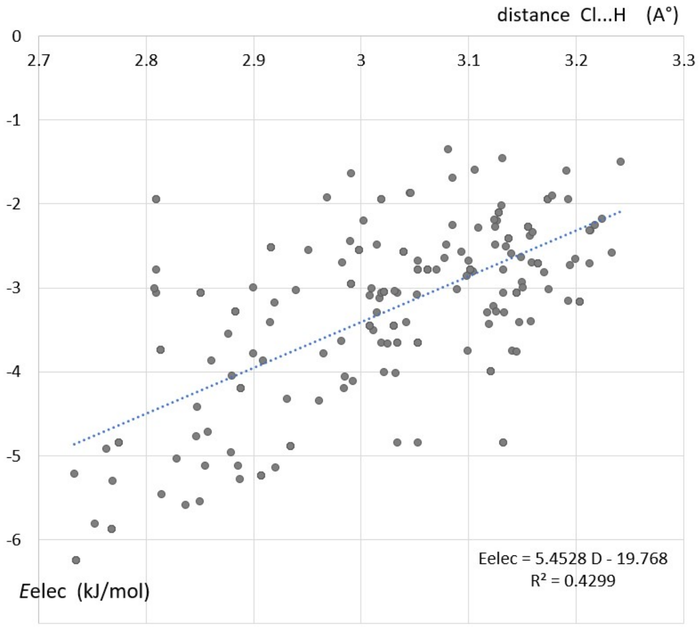

Figure S2. Scatterplot of E_{elec} vs. the Cl…H distances in the chlorinated aromatic hydrocarbons.

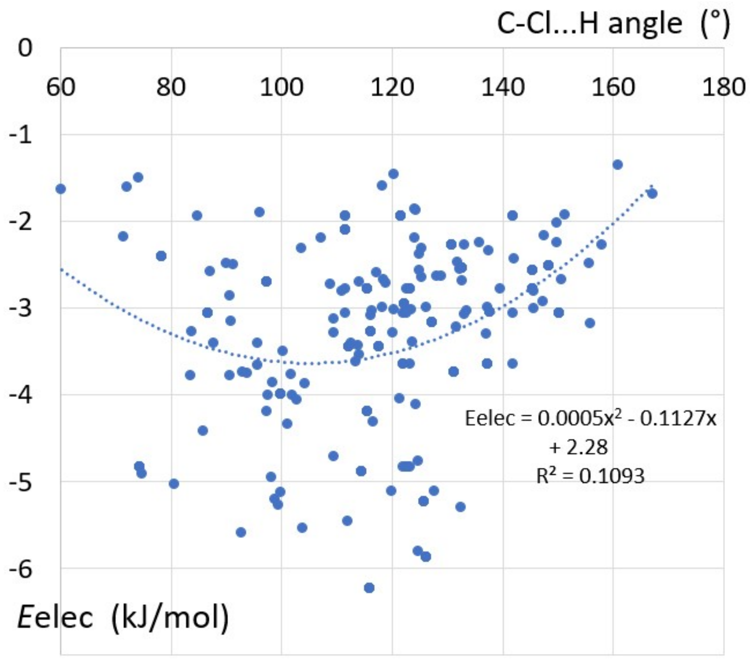

Figure S3. Scatterplot of E_{elec} vs. the C–Cl…H angles in the chlorinated aromatic hydrocarbons. The grey curve represents the frequencies for a uniform distribution in all directions.

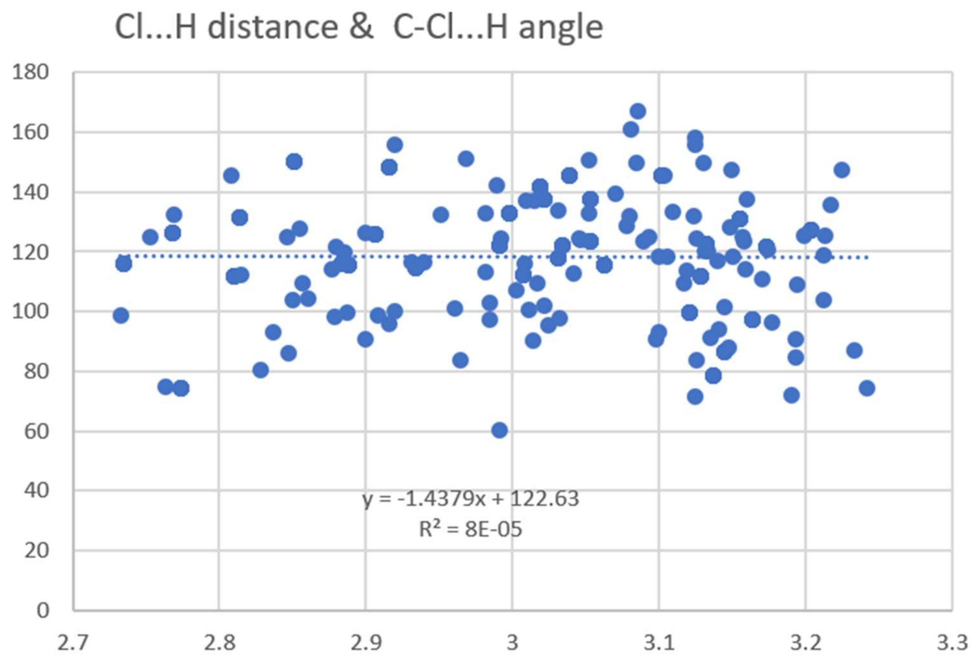

Figure S4. Scatterplot of Cl…H distance vs. the C–Cl…H angles in the chlorinated aromatic hydrocarbons

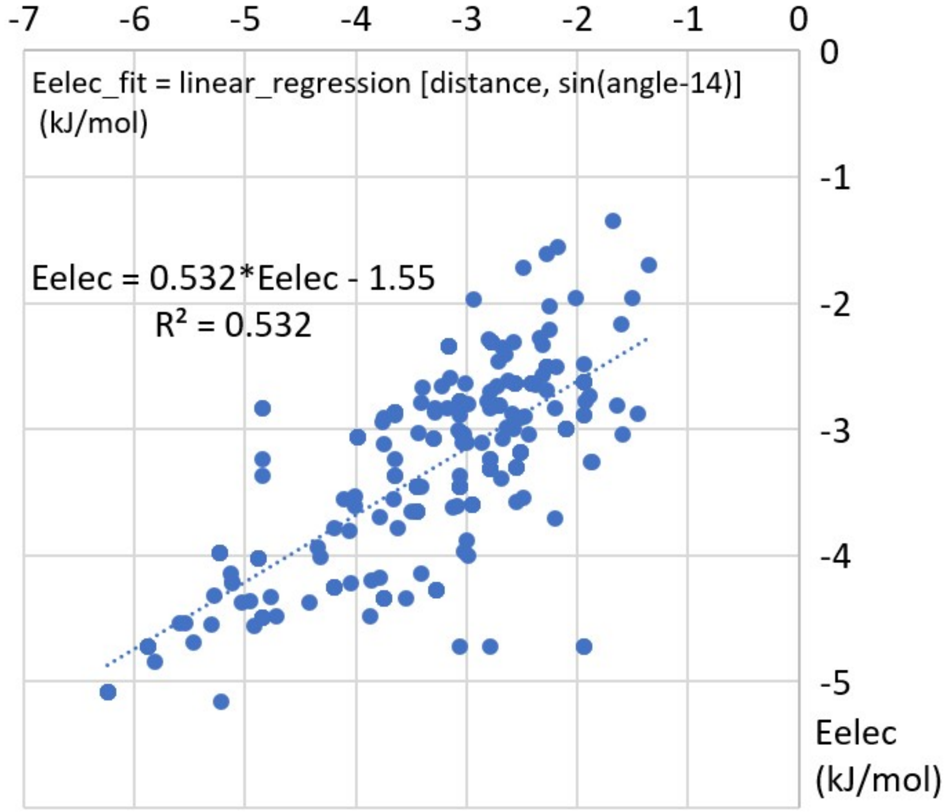

Figure S5. Scatterplot of E_{elec} vs. linear fit of E_{elec} of Cl…H interactions in chlorinated aromatic hydrocarbons. The best linear fit of type using the distance d(Cl…H) and sin[angle(C-Cl…H)-θ] was found for θ=14°. E_{elec_fit} = 5.4070 * d - 3.475 * sin[angle-14°].

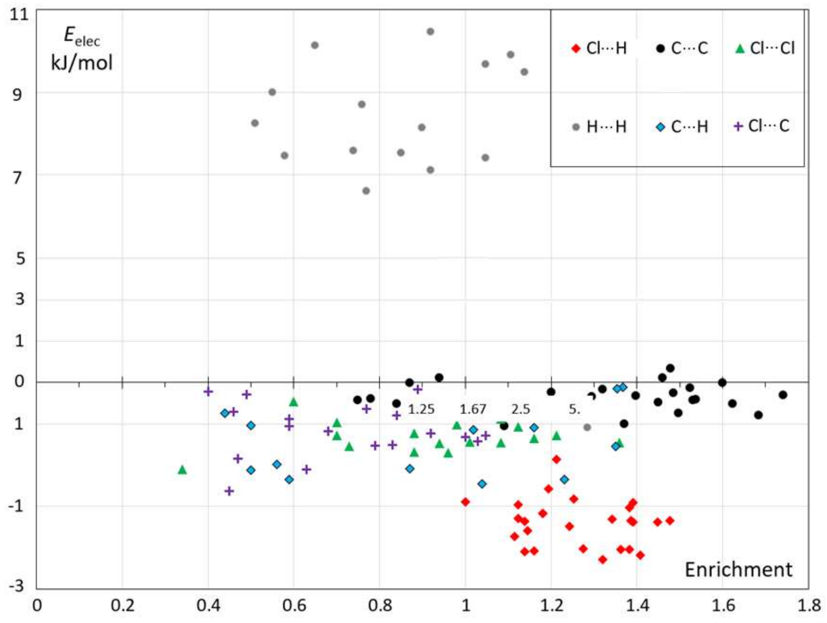

Figure S6. Scatterplot of individual enrichment and electrostatic energy for the different contact types in chloro-hydrocarbon molecules.

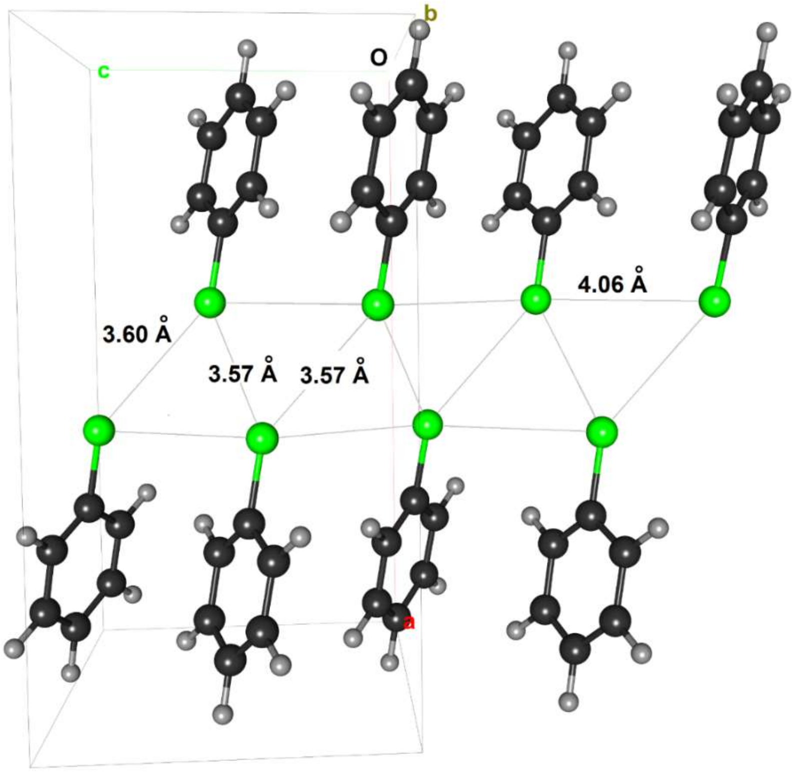

Figure S7. Crystal packing of chlorobenzene compound (MCBENZ). View of a quadruplet of interacting chlorine atom which form a chain along the **c** axis.

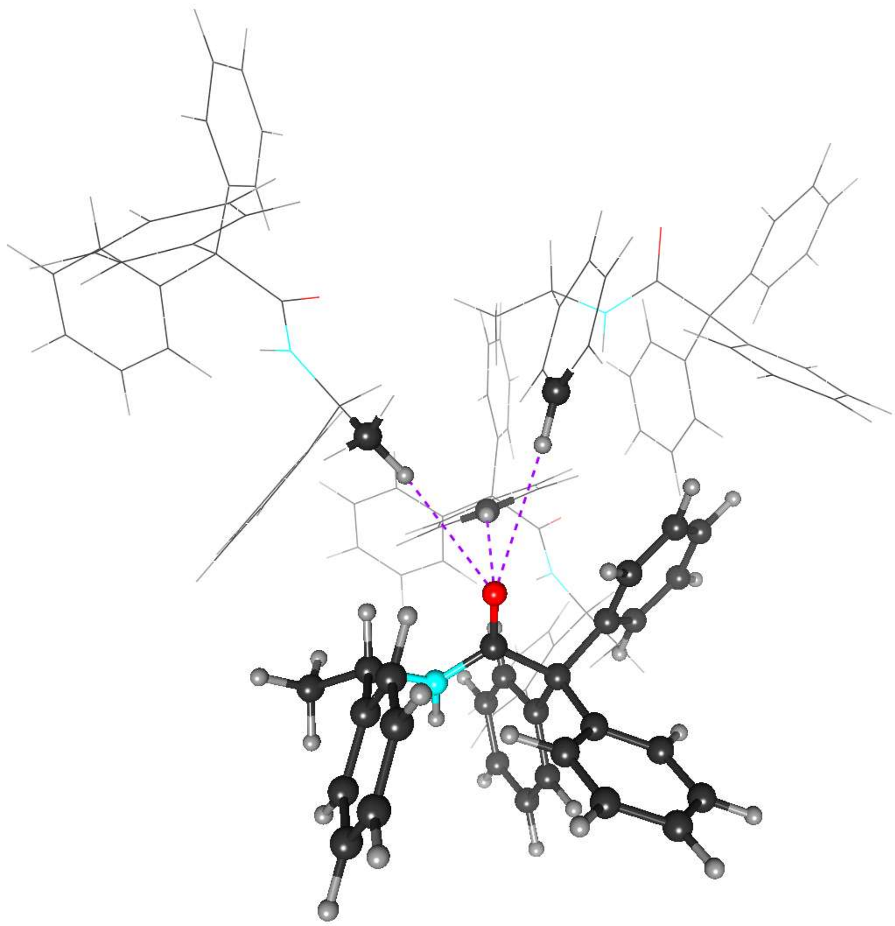

Figure S8. View of the RODJUP amide molecule crystal packing. There is no strong C=O…H–N hydrogen bond but an interaction of the amide oxygen with three C–H groups.

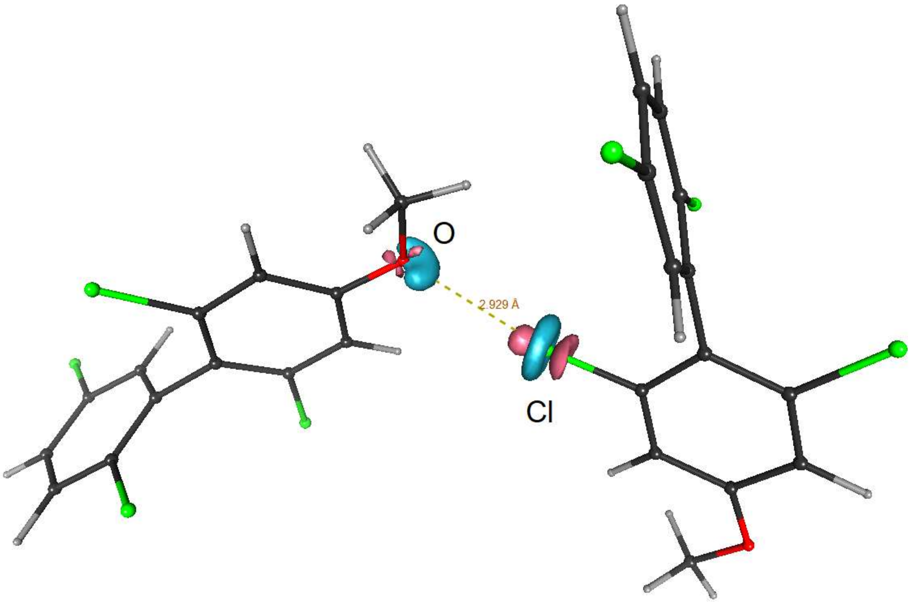

Figure S9. View of the only Cl…O halogen bond found in chloro-ethers with a negative E_{elec} value (-2.1 kJ/mol) for compound EBENIL in the CSD. The modelled electron deformation density is shown on the interacting atom, contour 0.2 e/Å^3, blue: electron accumulation, pink: electron depletion.

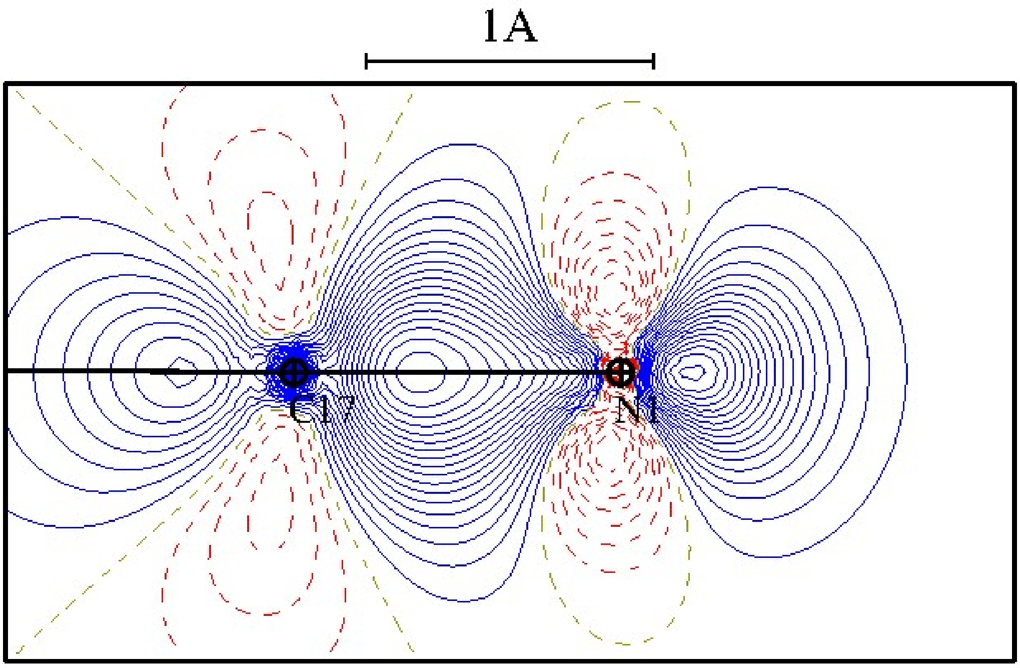

Figure S10. Deformation of the electron density of the –C≡N group, after transfer from the ELMAM2 database of multipolar atoms. Contour +/- 0.05 e/Å^3. Electron accumulation: blue, depletion: red. Zero contour in green.

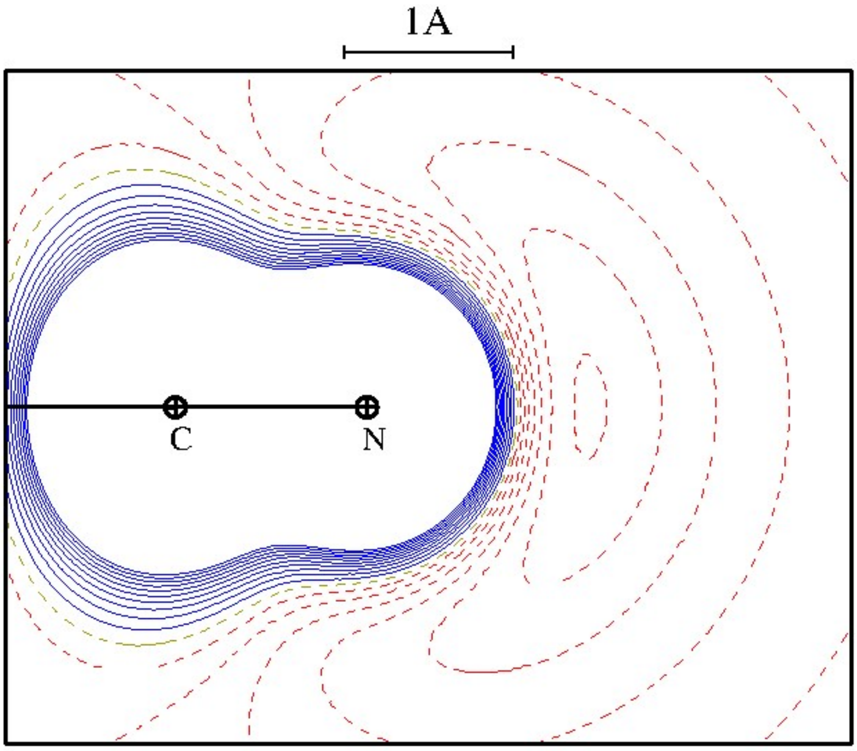

Figure S11. Total electrostatic potential generated by the –C≡N group after ELMAM2 database transfer. Contour +/- 0.05 e/Å. Electron accumulation: blue, depletion: red. Zero contour in green.

List molecules selected in the CSD.

Aromatic hydrocarbons.

BCYBUE01 BENZEN BUTBNZ CEKWEU COPMUR DIBENZ04 DIPRBZ DITBOX DURENE FLUNREN01 GOHWAB HAYXOU HBZPEN HMBENZ04 MEPRBZ MEYFIH MEYFON MEYFUT MHXBEN VAJHAP ZZZITY01 ZZZSPY01

Alcohols

AFILEI ASOCER ASOTOR BCHMOL BESWON BEVBAF BILMIU BOMKUJ CAVDEI CEJXIY CERMOB CERMUH CYOCDL01 DABKOG FECCOF01 FEPKUG FERTAY FEXKOI FIFRIV01 FUNCIA GIGYAW GOXMAH GUHBIU01 GURBUQ GURBUQ01 HEBDIB HIYHAY HOCCAD HUKLIJ HXCHEX ICAHID IQAFAI JAJSOC JEDBUQ JEQZUB KAPNAQ KEKPEW KEZTIU KEZTOA KUKYAR KUXFIT LOJXIS LONGAX MAMGEN MATLIC MENVIL MURABD MUTYOP NEPGCL01 NEZFON NIWNUC NULZEA NUMTUK PELBIR PERYTO04 PICYAB PILRAE PIMARB PIRBOJ PIRLOT PIWXIK PIYXOL PIYXOL01 POHYEQ POJGUQ PUFJIJ QATTEK QATTOU QATVEM QAZQAJ QIMCUK QURRUQ QUTFOA RAJDIP RIHMAX RIWTAT01 RIWTEX01 RIWTIB01 RIYZEF ROGNIL RUSKEW RUVWAG SILGEZ SUPMIA SUPMOG TIQHAC TIXXED VAHXAD VIWHAL VIWTAX WEFWAH WEJMAA WERTUI WERTUI01 WESTET WESTIX01 WOYKOK XEYMIX ZEFPAD ZINWAU ZZZFFY01 ZZZGSC01 ZZZKPE01 ZZZPSA01

Esters

ALUVIM BEFJUR BOBCIE CINGAH DOJFUF FEJKOV GEJLAK IWUFAH JORJEF JORJEF01 KAMRAS KEJXII LITLIK MAMNAC NIFCUA OMIDOD PATCUI PEHWAA QETVUG SEJWOT SUNHIS TELREH TIHNAZ TORMAP VESCAY VESDAZ VOHNAH WECCAI WECCUC01 WIRPAO XEGKID MAJYAX BOJZUV DODCHD FAFDAS01 FAFDAS YARZUN03 VEXRAR LEDPIT PAPVOR NUQPIZ HINHUJ BOPVIN JAXMIF FAWLEU KUZXIN DOJGAM CORNIH MESQOR FUMJON FAFDAT TIHMUS YIXTUV TIVCOR FICYUM ECOTDA SEDLUI JEDZAT HOTBOI JIRXIS GOKMOK QIKBER PIYYIG KEJXOO FOLVUX HOSNAE CORZAK XODHAZ PERCEN PEKKEV SURCIR FITGEU XAGKEW NETDOG DADQIK PILLEB UCOJOM SIQQEP FATRUN GEQQIC ZILQAM LEDNOX NIFCOU SURCEN HEGSAN01 ZAMVAK DUFMIA ADUQIZ MTCDEC QOYKIY KAXPOP GOGXUV BOSPUO VANLEB WELSUD HINJEV NACCID HIFWOI

Chlorinated C6 aromatics

ABUMIT CTCLST DAWRUO DCLANT10 DCLBEN01 DCLBIP DUXRAP ELINUI FAHTEO FECZOE FIXWUE FUHDAN01 GUQYOG HCLBPH IRUYIE KASJIY LIYPOZ MCBENZ NEHZAD OVIRAN PNCLBZ POGNUU QQQFED02 SIFGIX TCHLBZ TCLBEN02 TCNAPH YAFHUI ZZZBLP01

Amides hydrocarbons.

AHEYOC AHINEK ALITOE APHAMA AZTCDO BERWEB BIBVOZ BOYHIH CAPLAC CAPRYL CAYVOO CAZPUQ CAZQAX CAZVEF CDBALA COJFIR COSYAK CUMJID CUMJOJ CUNSEK DIJGIN DNCYDO DOGTIC DZCDON EHABUL EMAHUV ENANOL EZONOW FAHQIP FENQAS FIHDOR FISNEA FUWTUN FUWVAV FUZMIX HOHLUM HOHMAT JEJKIS KIKHAO LAYHUP MAYWAK MEJMEU NAAIBA NAHBON NAKLOA OCIMBC OJESIF OJIBIS OJICAL PHETAC POLPIP RODJUP SUGYOJ TBPIPO TEKYOZ TEKYUF TMZCNO WOLXOK XAQHAZ XIYBIQ XOFTER YAZKOZ YOFNEN YUPHAS YUPHEW YUPHIA ZEVDAF ZOBXIX ZOBXOD

Amides + Pyridines

AHUBEM AROJAU ATAYOK AVURAK AWUNOV BILXUR CEGMOS CEJPUC CICTUE COFJEO CUBRIC DIFMIQ DOPDAN DOWROY DUXCUV EDAXOW EHAHEC EHIPOA FADZIV01 FAFBAR HALQER HEJMER HEYMII JEPMAS JOFWIM01 LABDIE LAMDIO LIJPAW LIVQAK MOHQOP MURWUR NOQYUP OFOWOW01 OMODAV PAVSEM PEDCUX PEDDIM PETBOH PETBUN PILMON PYDCXA10 QUCXMA11 ROFGUP SIBCAI SIVCAB01 SUNVUT SUZHEB TIHHID TITSUL TITTIA TITWEZ USOFIT UTAQEN UTESIX UTUXIS UXEYAY UXEYOM VASGIH VASHII VASHOO VEVDUW VUCVIY VUCVOE VUJVIG WOVYAH WUXQOW XIFNIK01 YAVWUO YIRMES YUPVUB ZAVWAU

Chlorinated ethers:

KARJES DCPHER FIDBUR MTCPXP YIKJAF AZOPEL BONDEP FAMFOO FUDBAI HUGBAN LOCBEM MILPUT MUWROM NAVGAU ODOTUW ODOVEI UDANAP VAFBEJ YAWLIT AZAPAS AZAPEW DAGPAD DAGPEH EBENIL EZITEM FELSEU GEKWOI GEKWUO GISXEM IDIWUP IRUYOK IRUYUQ JAHTOB MEMXIM MUDVIS OGODAR OKEGIV QEZRES QOTDIN RAXYIA RISDED RUSGAP SENJUR SENKIF SESHED TARZIW UZIMEX XOZJIF XUBNUF YEBGUH YUPWAI

Fluorinated nitrile hydrocarbons

ABAKIZ AWODOH DAHXAO EFATOU LONTIS PONLOU QACLOV QOSBAC SIKREJ TEBBIL UBOZUJ UCOJOL UFAZOQ VEVWUQ VEVXAX WAJVUZ XAKJAX YOHMAL ZIZYEO

ROY polymorphs

ON: QAXMEH Y: QAXMEH01 QAXMEH02 OP: QAXMEH03 YN: QAXMEH04 YT04: QAXMEH12 QAXMEH53 ORP: QAXMEH55 QAXMEH59 R05: QAXMEH31 (dimer in asymmetric unit.)
